# Reinventing the spermatheca: unveiling a novel sperm storage organ in Epilachninae ladybirds

**DOI:** 10.1098/rsob.240395

**Published:** 2025-06-25

**Authors:** Paulo Henrique Rezende, Glenda Dias, Mauricio da Silva Paulo, Dayvson Ayala-Costa, Ana Clara Pereira Teixeira, José Lino-Neto

**Affiliations:** ^1^ Departamento de Biologia Geral, Universidade Federal de Viçosa, Vicosa, Minas Gerais, Brazil; ^2^ Departamento de Entomologia, Universidade Federal de Viçosa, Vicosa, Minas Gerais, Brazil

**Keywords:** ladybugs, Coccinellidae, insect reproductive system, seminal receptacle

## Abstract

This study investigates the reproductive apparatus of *Epilachna clandestina*, focusing on its secondary spermatheca, first described as a ‘seminal node’ by Katakura *et al*. for the Asian Epilachninae. We corroborate their previous findings that highlight this organ as the primary sperm storage site, contrasting with a vestigial typical spermatheca that lacks significant functionality. The functional spermatheca is a dilated, secretory region of the common oviduct filled with sperm. It features a specialized epithelium containing class 3 secretory cells and is lined by a thin chitinous cuticle, indicating that it qualifies as a true spermatheca. This organ seems to optimize sperm storage and maintenance when compared to the vestigial spermatheca. Additionally, we emphasize the close association between sperm and epithelial structures as mechanisms for efficient sperm retention and dosage, which could quantitatively and qualitatively improve reproductive success. Our findings may contribute to a greater understanding of the evolutionary adaptations of reproductive structures in Epilachninae and raise questions regarding the evolutionary history of these organs within ladybirds.

**Figure:**
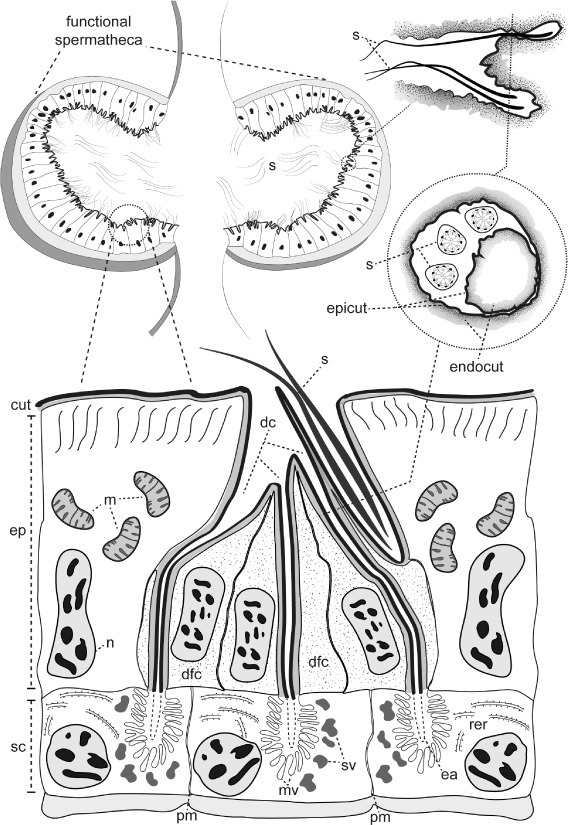

## 1. Introduction

What is a spermatheca? The definition is that it is a sperm-storing sac-like organ in several female organisms, mainly insects [1,2]. That is its function, but what about the morphology of this organ? In insects, it has an ectodermal origin. It usually comprises a reservoir of a simple epithelium supporting a storing chamber lined by a thick chitinous cuticle, and a spermathecal gland often accompanies it [2]. Other times, there is no separate gland; glandular cells are part of the epithelium. In either case, the glandular cells can be classified usually as class 3 according to their organization and morphogenesis [2–7]. Non-insect hexapods, such as Collembola, also possess true spermathecae, lined by a chitinous cuticle, and present spermathecal accessory glands, although both organs have type 1 gland cells [8]. Diplurans also seem to have this organ [9], but a detailed morphology is not described. There are no available data on Protura at this moment. Other organisms such as arachnids [10], molluscs [11], annelids [12], nematodes [13] and amphibians [14] also have organs called spermathecae; however, in the non-arthropod groups, this structure does not contain a chitinous cuticle. Across all these groups, these organs possess wide morphological variations, reflecting their particular reproductive strategies.

Some insects, such as flies, possess two or more spermathecae [2,15]. On the other hand, some other insects have lost the typical spermatheca, storing sperm in different organs, as in a bulb-like portion of the lateral oviducts, as in lace bugs (Tingidae) (and not in the pseudospermathecae as previously thought) [16], or as in bedbugs (Cimicidae), where they developed another sperm-storing structure called *seminal conceptacle* [17,18], in those latter two cases, having mesodermal origin. The term spermatheca is not employed for these bugs, as in other insects with sperm receptacles with similar morphology not homologous to the ectodermal spermathecae. The Epilachninae ladybirds (Coccinellidae) were described by Katakura *et al*. [19] as having a typical spermatheca but additionally showing a budding or dilatation on either side of the medial common oviduct, which seemed to be storing sperm instead of the usual organ. The authors named this organ a ‘seminal node’ while calling the first structure a ‘vestigial spermatheca’. We observed the same finding in the ladybird *Epilachna clandestina* Mulsant, 1850 (Coleoptera: Coccinellidae) and selected this species for thorough analysis. We described their reproductive system using light microscopy and transmission electron microscopy (TEM), focusing on their spermathecae to elucidate their morphological nature. We discuss our findings and possible implications for their reproductive biology and propose an update in the terminology for this sperm-storing organ in this subfamily of ladybirds.

## 2. Material and methods

### 2.1. Insects

Adult females of *E. clandestina* were collected from *Sechium edule* (Jacq.) Swartz, 1800 plantations at the Campus of the Federal University of Viçosa, Viçosa, MG, Brazil (20°45′14″ S, 42°52′55″ W, 648 m).

### 2.2. Anatomical analysis of the reproductive system

For anatomical analysis, 10 individuals were cryo-anesthetized, and the reproductive systems of the females were isolated in phosphate-buffered saline, pH 7.2 (PBS), freshly fixed in 2.5% glutaraldehyde buffered in PBS for 3 minutes. Then, they were placed on hollowed-out glass plates with drops of the same buffer. The reproductive system was photographed unstained with a camera (Nikon D200) attached to a stereomicroscope (Zeiss Stemi 2000-C).

### 2.3. Histological sections

The region of the common oviduct containing the spermatheca was isolated after dissecting the females in PBS. It was then transferred to a fixative solution of 2.5% glutaraldehyde in PBS with 3% sucrose for 2 hours. The material was washed in the same buffer, post-fixed in 1% osmium tetroxide for 2 hours and dehydrated using alcohol solutions with increasing concentrations: 30, 50, 70, 90 and 100%. Then, the material was immersed in two 4-hour baths, each at ambient temperature, the first with a mixture of historesin (Leica Historesin, Heidelberg, Germany) and alcohol (1 : 1) and the second with pure historesin. For inclusion, they were immersed in historesin with a catalyst in silicone moulds, which were placed in Petri dishes and transferred to an oven at 58°C for 24 hours. Semi-thin sections (0.5 µm thick) were obtained with glass knives with a microtome (Leica RM 2155, Leica Corporation, Wetzlar, Germany). These were transferred to histological slides stained with Harris hematoxylin (Merck, Darmstadt, Germany) for 15 minutes, washed in running water for 10 minutes, stained with eosin (Sigma-Aldrich, USA) for 1 minute and rapidly rinsed in tap water (see [20]). All observations and photographs were made using an Olympus BX-60 microscope (Olympus Corporation, Tokyo, Japan).

### 2.4. Transmission electron microscopy

Females of *E. clandestina* were dissected in PBS, and their reproductive systems were fixed in 2.5% glutaraldehyde in PBS added with 3% sucrose, for 24 hours at 4°C. After this period, the samples were washed in distilled water and post-fixed in 1% osmium tetroxide. Then, the materials were subjected to a new wash and dehydrated in an alcohol series (30, 50, 70, 90 and 100%) and acetone. Once dehydration was complete, each sample was infiltrated and embedded in epoxy resin (Epon 812). Ultrathin sections (approx. 60 nm thick) were obtained with an RMC Products Power Tome-X ultramicrotome and counterstained with 3% uranyl acetate and 0.2% lead citrate solutions. All samples were observed and photographed using a Zeiss EM 109 transmission electron microscope operating at an electron acceleration voltage of 80 kV.

## 3. Results

**Figure 1.**
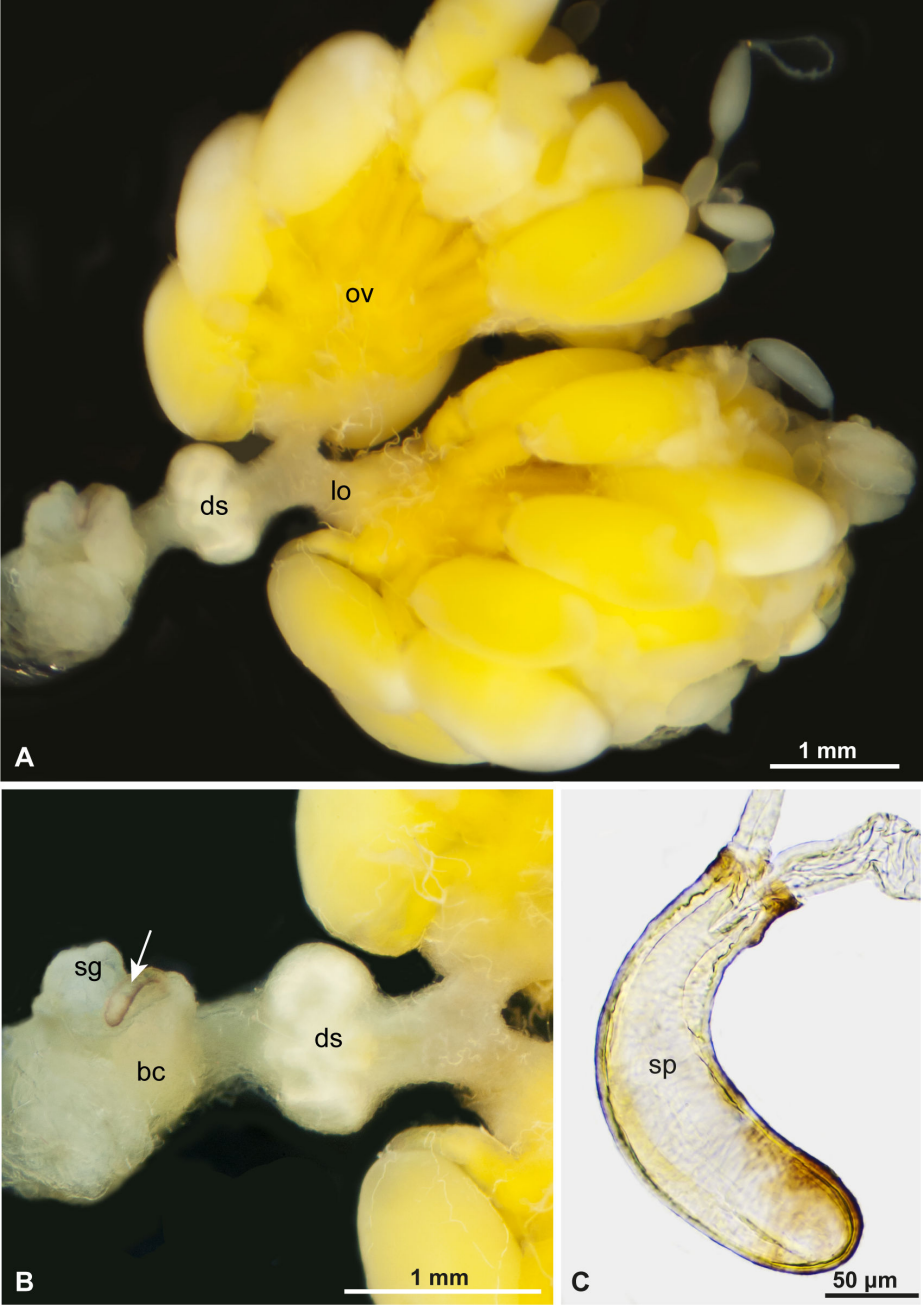
Female reproductive systems (A,B) and spermatheca (C) of *Epilachna* sp. Ovary (ov), lateral oviduct (lo), common oviduct (co), dilatation with sperm (ds), bursa copulatrix (bc), spermatheca (sp), and spermathecal gland (sg). Note (in B) the difference in volumes between the spermatheca (arrow) and the dilated region of the common oviduct (ds).

**Figure 2.**
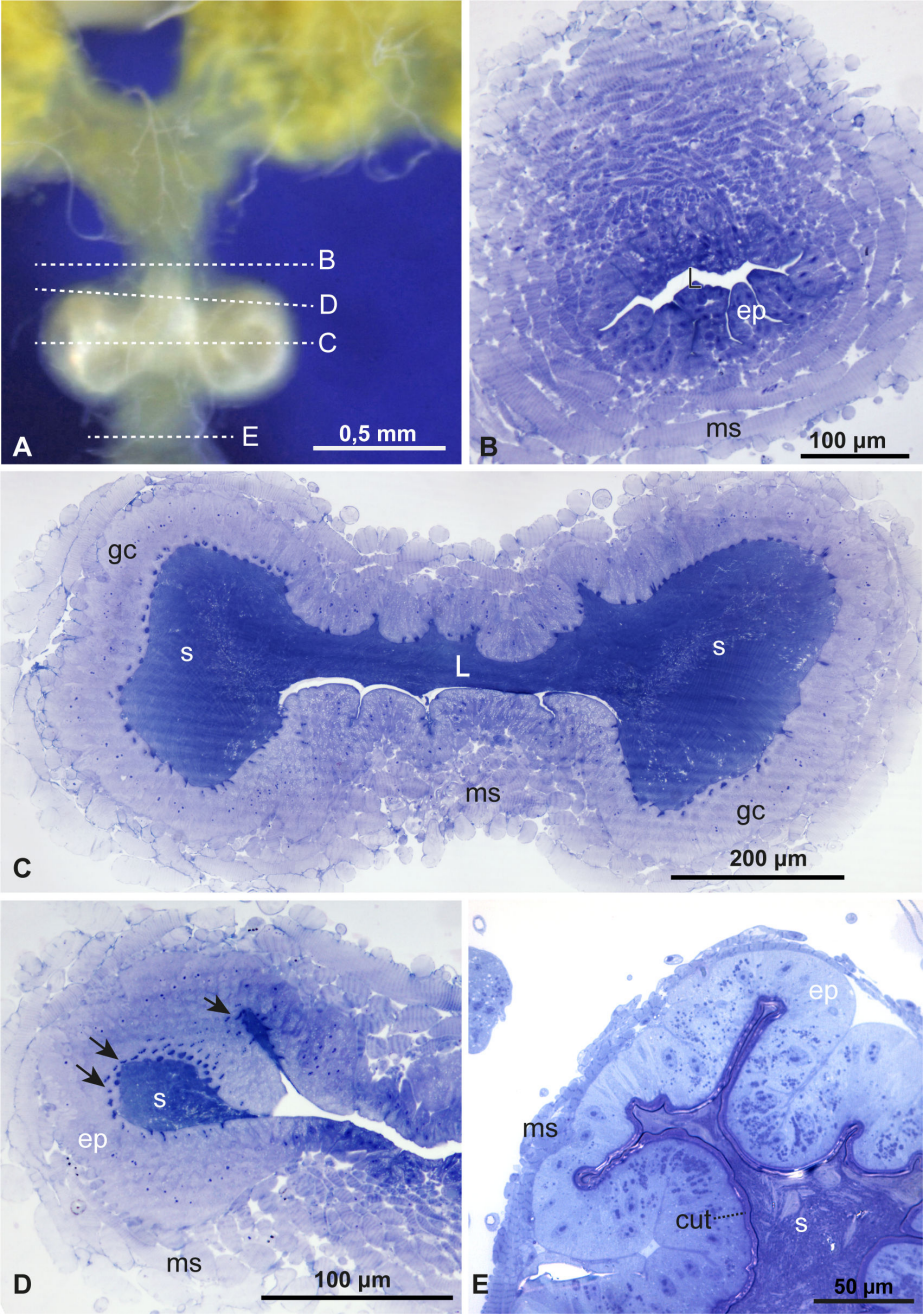
Functional spermatheca under light microscopy. (A) Orientation scheme of transversal sections of the functional spermatheca. (B) The distal portion of the common oviduct, with a thick muscular layer (ms), the epithelium (ep) has slightly elongated cells and the lumen (L) is narrow. (C,D) Section of the functional spermatheca at different heights. The lumen (L) is filled with spermatozoa (S), and the epithelium has many glandular cells (gc) and glandular ducts (arrows). The muscular layer (ms) is thick in this region. (E) Histological section of the common oviduct in the distal portion of a recently mated female. It shows plenty of spermatozoa (S) in the lumen and an epithelium (ep) with smaller cells and a thick cuticle (cut). The muscular layer (ms) is thinner in this region.

The female reproductive system of *E. clandestina* includes a pair of ovaries, a pair of lateral oviducts, a common oviduct and two types of sperm-storing organs: one functional and the other vestigial (figure 1A–C). Each ovary consists of six to eight ovarioles connected to the calyx of each lateral oviduct. In the medial region of the common oviduct, there is a thick, bilobed structure where sperm are stored, the functional spermatheca (figures 1B and 2A). The typical (but vestigial) spermatheca is a short tube connected by a narrow duct to the common oviduct at its basal portion (figure 1B,C). This organ is highly chitinized, and its lumen was devoid of sperm in all dissected females (figure 1C). A small tubular accessory gland accompanies the vestigial spermatheca. In contrast, the functional spermatheca does not have a separate gland.

### 3.1. The functional spermatheca

In histological cross-sections, the functional spermatheca (figure 2A) displayed a bilaterally dilated region, with areas on both sides filled with sperm (figure 2C,D). However, even the lumen of the distal common oviduct contained sperm in recently mated females (24 hours after mating observed in the laboratory) (figure 2E). The spermathecal epithelium comprises elongated cells with a slightly elongated nucleus of uncondensed chromatin, evidencing numerous nucleoli. Furthermore, the epithelium is lined by a thin cuticle, which delimits the sperm storage area filled with spermatozoa (figure 2C,D).

**Figure 3.**
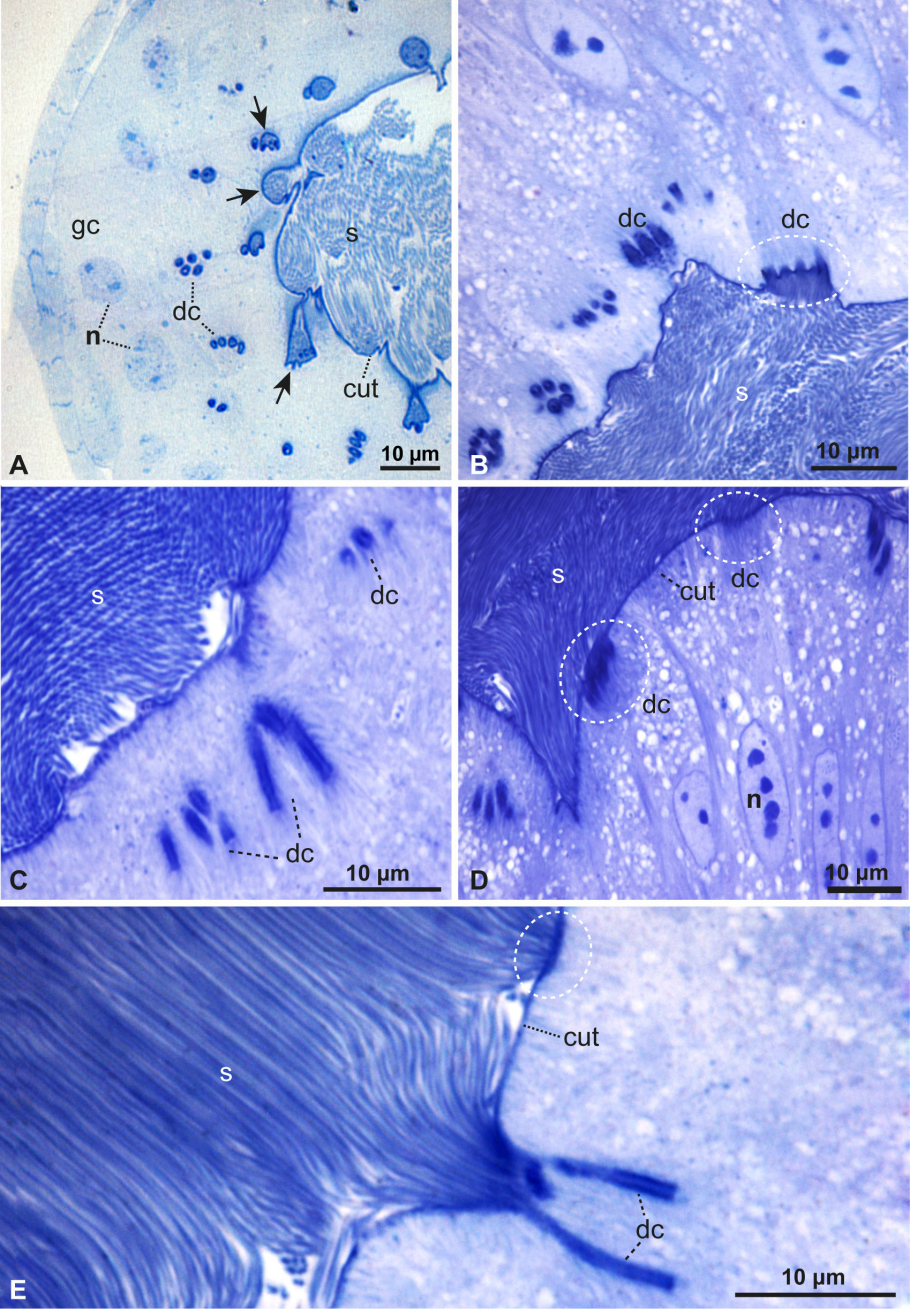
Functional spermatheca under light microscopy. (A–E) Details of the dilated region showing glandular cells (gc), spermatozoa (s), glandular duct cells (dc), the nucleus of glandular cells (n) and cuticle (cut). The dotted circles and arrows show the region of fusion of the glandular ducts with the cuticles.

A thick layer of muscular tissue surrounds this spermathecal region, with muscle cells oriented vertically and horizontally (figure 2B,D). The lumen where sperm are concentrated shows epithelial invaginations where secretory ducts seem to connected (figure 3A,B,E). Also, this lumen is bordered by a layer of highly vacuolated glandular cells. In small clusters, the ducts of these cells can be observed opening into the invagination pockets of the cuticle lining the lumen along the entire epithelium (figure 3B,D). A peculiar finding is that the sperm heads are inserted into the epithelium in invaginations of the cuticle that give rise to narrow canals where sperm can enter (figure 3A–E).

**Figure 4.**
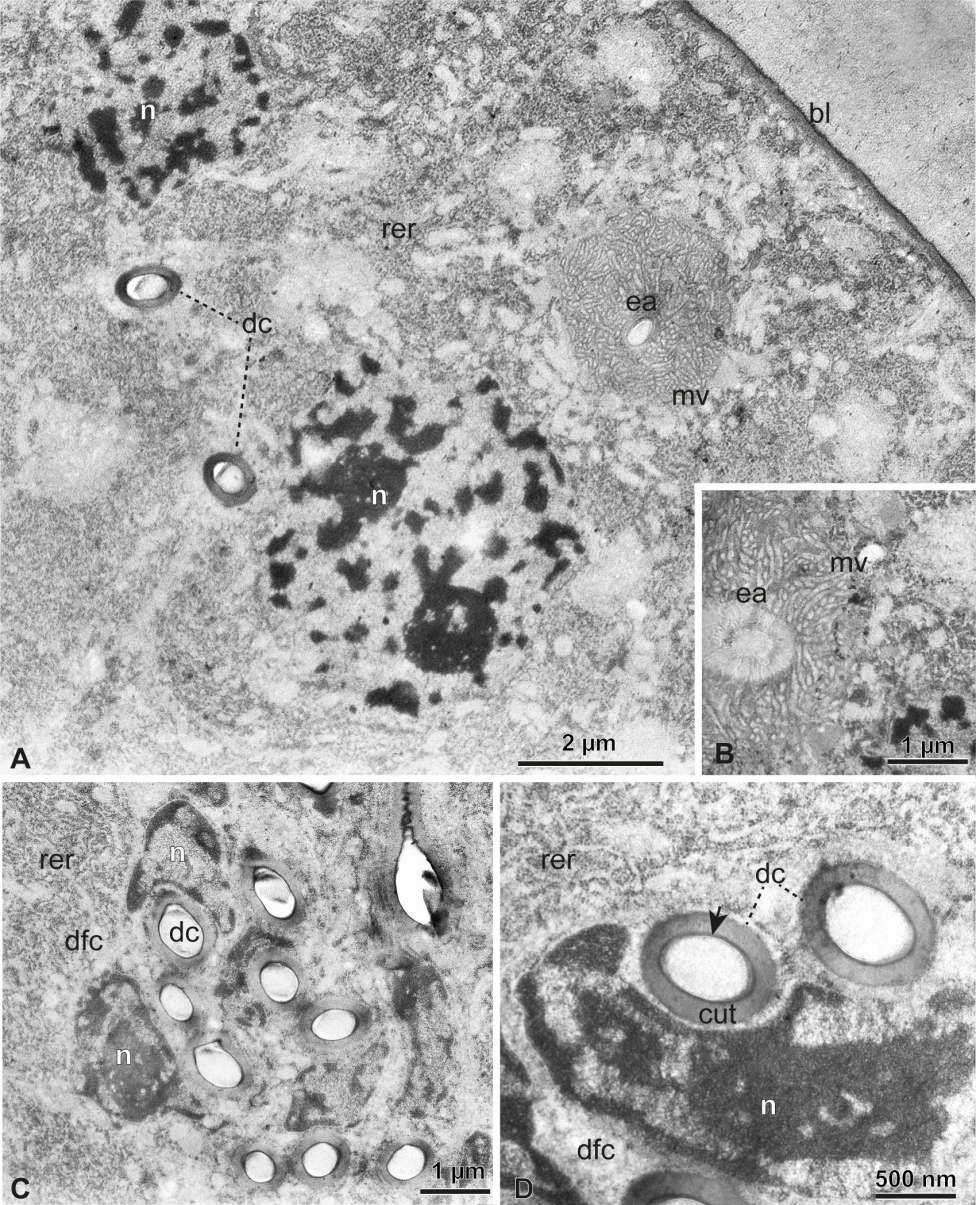
Functional spermatheca under TEM. (A) Epithelial cells have nuclei (n) with predominantly decondensed chromatin and evident rough endoplasmic reticulum (rer). Ducts of glandular cells (dc) are observed between the cells and the end apparatus (ea) with its microvilli (mv) (as in the inset in B) located close to the basal lamina (bl). (C,D) Nuclei (n) of duct-forming cells (dfc) with different levels of chromatin compaction. Several ducts (dc) with thick cuticle (cut) with a darker epicuticle (arrow) are observed.

**Figure 5.**
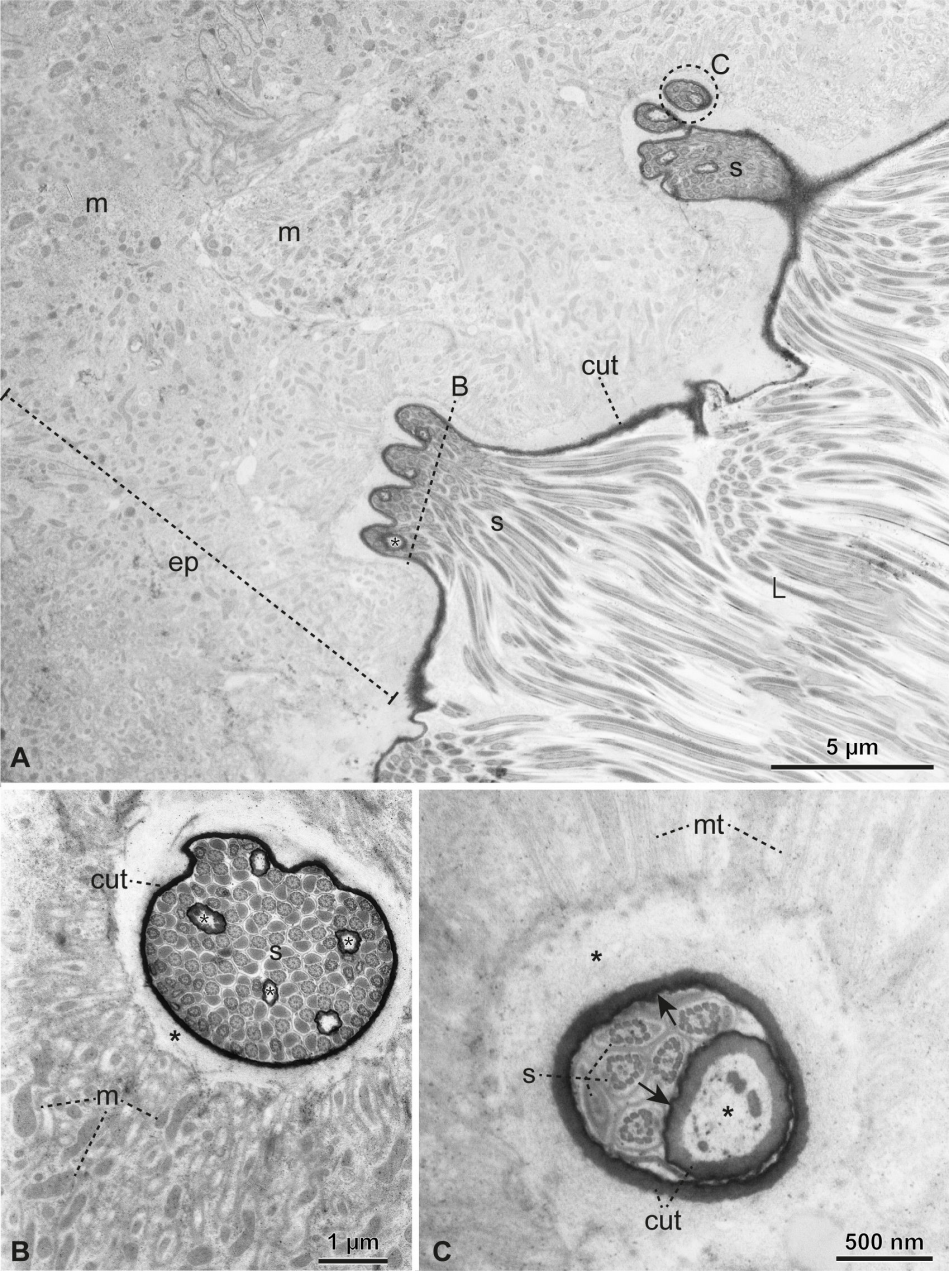
Functional spermatheca under TEM. (A–C) Association of sperm (S) with epithelial pockets and glandular ducts. In this region, the cuticle (cut) is thin. The epithelial cells (ep) have many mitochondria (m) and microtubules (mt). Note in the cross-sections in (B,C) that the secretory ducts merge with the invaginations of the cuticle that lines the spermathecal lumen (L), forming pockets filled with sperm (S). (C) The secretory ducts fold inward, as evident by the inside-out epicuticle–cuticle arrangement (arrow). Asterisk: subcuticular space.

**Figure 6.**
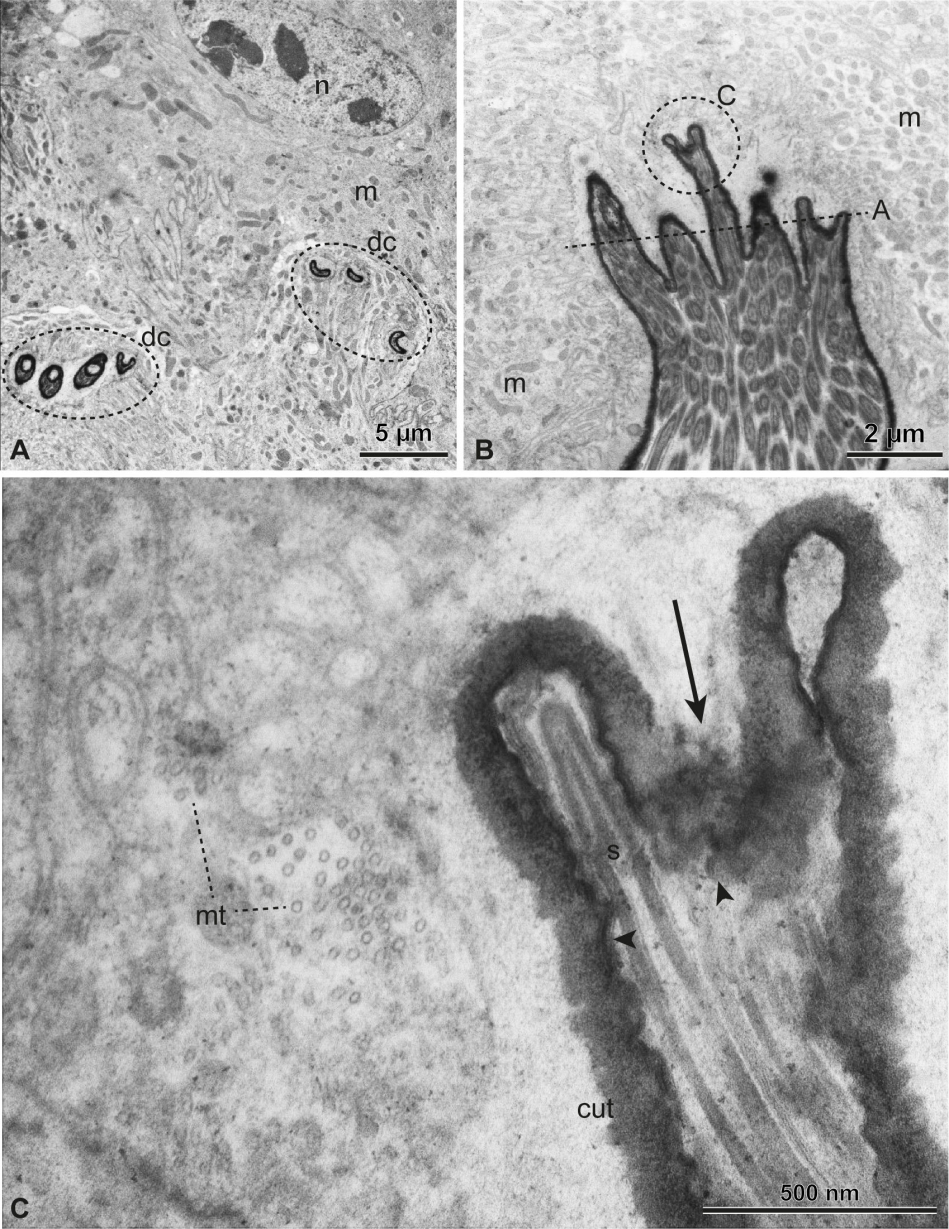
Functional spermatheca under TEM. Transverse (A) and longitudinal (B,C) sections of the ducts of glandular cells (dc). The nucleus (n) is evident in (A), with decondensed chromatin and many mitochondria (m). In (B,C), detail of the invaginations of the epicuticle–cuticle (arrowhead and cut), with ducts filled with sperm (s). Microtubules (mt) are also observed.

Under TEM, we observed the functional spermatheca surrounded by two or three layers of concentric muscles, as seen in histological sections. The epithelium is tall and has several class 3 glandular cells (figure 4A–D). The secretory cells are localized in contact with the basal lamina and show large spherical nuclei (approx. 12 μm in diameter) with quite evident nucleoli, and the cytoplasm is rich in rough endoplasmic reticulum (figure 4A). There is evidence for the presence of end apparatus in the secretory cells, that display microvilli pointing into the efferent ducts, penetrating the secretory cell (figure 4A,B). These ducts seem to be continuous with the cuticular secretory ducts (700–800 nm wide, with a single cuticular layer near 170 nm thick) supported by duct-forming cells (figure 4C,D). These latter cells have elongated nuclei, supporting a duct, and these form complexes of three to six ducts near the apex of epithelial cells (figure 4C). The secretory ducts seem to connect with the cuticle that lines the spermathecal lumen, that show infoldings, forming pockets into the epithelium (figures 3A, 5A and 6B). Also, something peculiar was the secretory ducts that seem to fold inwards, looking like a duct within a duct but the inner duct is inverted (figure 6C), as evident for arrangement of the cuticular layers (inside-out epicuticle–cuticle), opposite to the regular secretory ducts surrounding these infoldings (figure 5C).

Epithelial cells support the single cuticular layer (115–370 nm thick) lining the spermathecal lumen (figure 5A). They are rich in mitochondria (figures 5A,B and 6A,B) and a few cytoplasmic microtubules pointing towards the lumen can be seen in some points (figures 5C and 6C). A subcuticular layer around 500 nm thick is observed devoid of cellular structures and is also present around the secretory ducts near their merging point with the luminal space (figure 5B,C). We observed plenty of spermatozoa in the lumen, closely associated with the cuticular pockets. The sperm heads are inserted in them, and even inside the narrow secretory ducts that are continuous with these invaginations.

### 3.2. The vestigial spermatheca and spermathecal gland

**Figure 7.**
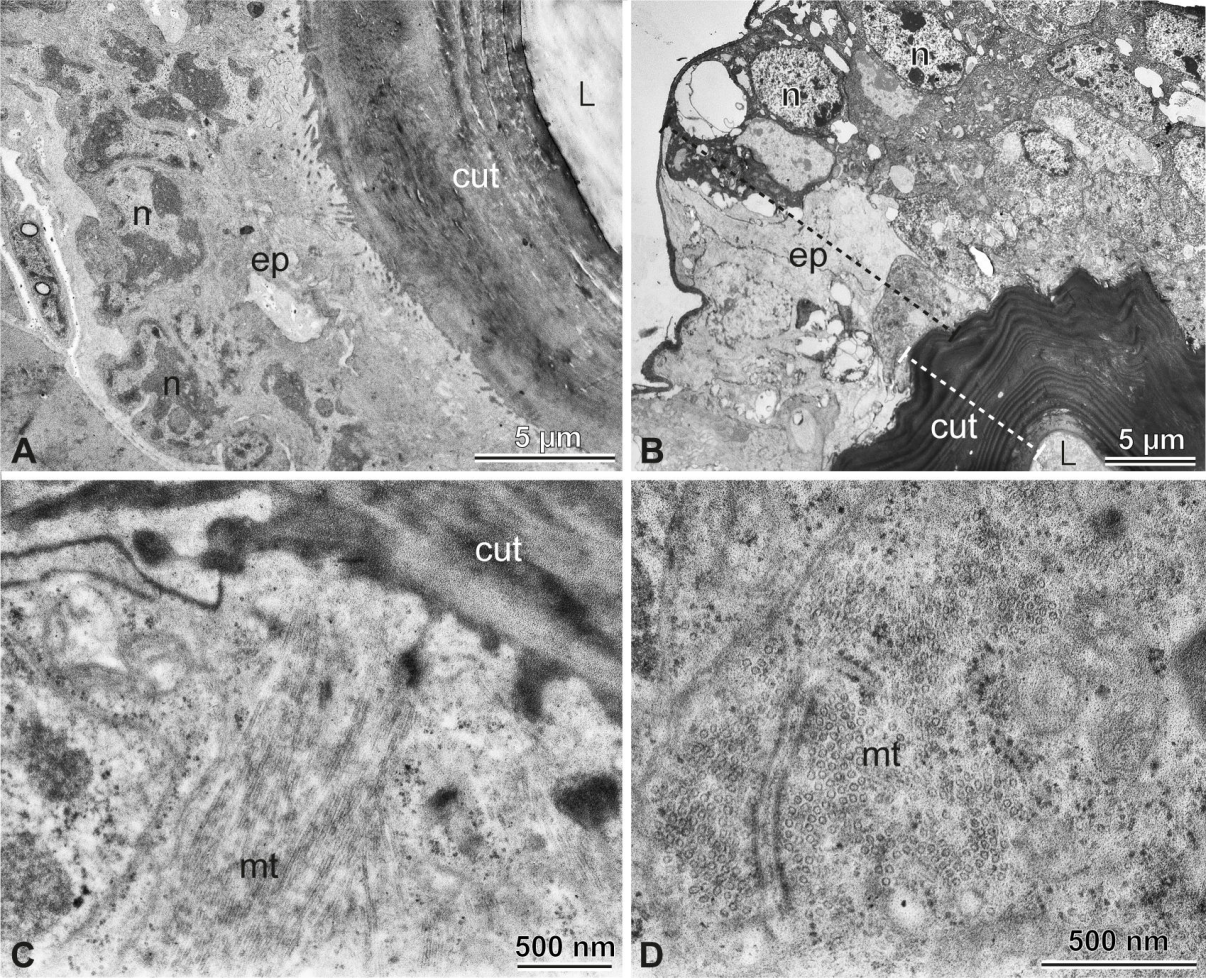
Vestigial spermatheca. (A,B) The epithelium (ep) of the vestigial spermatheca is simple columnar, with basal nuclei (n) and non-condensed chromatin. In the apical portion of the cells is a thick cuticle (cut), approximately 8 μm thick and 16 layers. No sperm was found in the lumen (l). (C,D) Microtubules (mt) in longitudinal and transversal sectioning, respectively.

The vestigial spermatheca is formed by a simple epithelium with columnar cells 12–25 μm tall (figure 7A,B). These cells showed few organelles and nuclei ellipsoid in shape with non-condensed chromatin and located close to the basal lamina. Their cytoplasm displays numerous microtubules pointing towards the basal part of the cuticle (figure 7C,D). The epithelium apical portion supports a thick cuticle that is approximately 8 μm thick and consists of about 16 layers (figure 7B). This cuticle outlines the spermathecal lumen, which we observed empty in all the copulated females studied.

**Figure 8.**
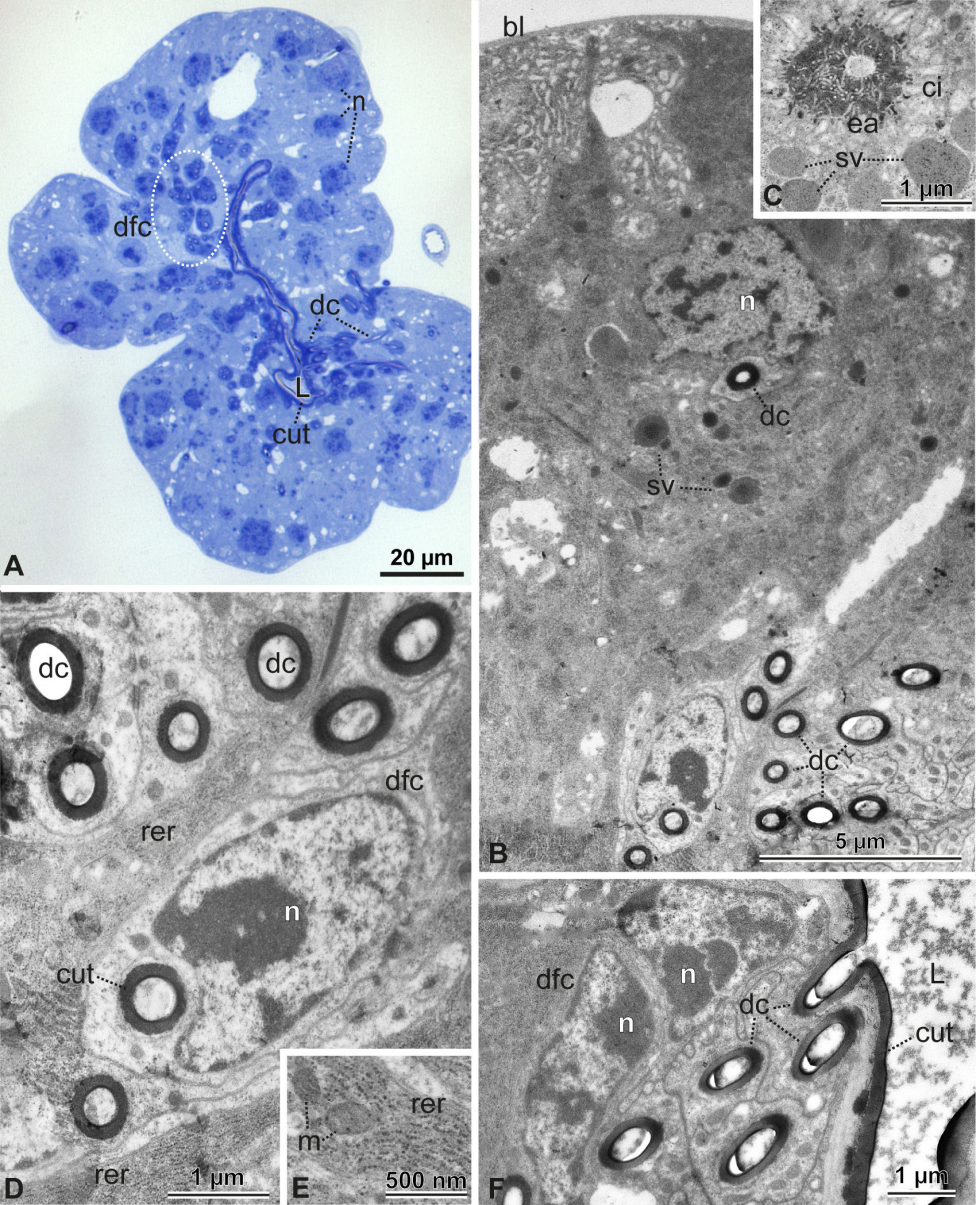
Spermathecal gland. (A) Cross-section showing the gland with an irregular shape. It is formed by an epithelium with type 3 glandular cells (dfc), with their secretory ducts (dc) merging with the cuticle (cut) and opening into the lumen of the gland. (B–F) The secretory cells are close to the basal lamina (bl), have nuclei (n) with decondensed chromatin, evident rough endoplasmic reticulum (rer), mitochondria (m) and secretory vesicles (sv). These cells pour their secretions into the extracellular space of the cistern (ci) lined by microvilli, and go through the end-apparatus and secretory ducts (dc). These latter ducts are supported by duct-forming cells (dfc). The secretions reach the lumen (L) of the gland at the point of fusion of the ducts of the cells (dc) with the cuticle (cut).

The spermathecal gland shows an irregular shape in cross-section (figure 8A). It is formed by an epithelium (21 to 35 µm thick) with many type 3 glandular units, with their secretory ducts opening into the gland’s lumen. The secretory cells are located in the basal portion and contain rough endoplasmic reticulum and secretory vesicles (figure 8B,D,E). These cells release their secretions into the cistern of the secretory cells (figure 8C). Many secretory ducts are observed and supported by duct-forming cells (figure 8B,C). These ducts transport secretions to the gland’s lumen and are connected to a thick cuticle that lines the luminal space (figure 8F).

## 4. Discussion

The female reproductive apparatus of *E. clandestina* resembles the regular pattern of insects [21]. It shows paired ovaries and lateral oviducts, the latter joining the common oviduct that connects with the genital opening and a highly chitinized vestigial spermatheca (and its gland) that flows into the oviduct distally. However, it also has a peculiar second spermatheca, called a ‘seminal node’ by Katakura *et al*. [19]. These authors noticed that the typical spermathecae were empty in all their analysed Epilachninae species; in contrast, the ‘seminal nodes’ were filled with sperm. Here, we confirm their original finding and add an in-depth morphological perspective to these organs.

Our study indicates that this region of the oviduct is the only sperm-storing organ in this taxon, qualifying it as a functional spermatheca. This medial region of the common oviduct is notably dilated and highly specialized for secretory activity, while the remaining portions do not exhibit such specialization. In addition to storing sperm, features such as the epithelium with type 3 secretory cells [3,4] and the apical cells lined by a chitinous cuticle (thus having an ectodermal origin) make us conclude that this organ meets the requirements to be classified as a true insect spermatheca [2,8], albeit a secondary one. Being so, we propose it to be termed a ‘functional spermatheca’. This organ seems to optimally replace the functions of the typical spermatheca that has become vestigial in Epilachninae; it can store a much larger volume of spermatozoa while having a secretory structure and functionality like a spermathecal gland. These secretory cells may play an essential role in sperm-supportive functions [22]. This structure is similar to the spermatheca of the beetle *Deronectes incospectus* (Dytiscidae), which also has a combined sperm storage function and secretory activity, with epithelium with class 3 secretory cells [23]. Either way, they seem more efficient than a typically arranged spermatheca of other insects [2,15]. This is not the first record of a female insect with two types of sperm-storing organs. Drosophilids (Diptera) have two typical spermathecae plus a long tubular seminal receptacle [24]. In these insects, sperm are stored in both spermathecae, but sperm from one are more likely to fertilize the eggs [25]. This observation led us to wonder whether, in the evolutionary history of Epilachninae ladybirds, it may have also happened, and later, the secondary spermatheca took the lead, becoming the only functional organ.

We also raised questions about the possible disadvantages of storing sperm in the oocyte passageway, which could carry away many sperm cells when laid. However, the secondary spermatheca seems to contour this problem by a peculiar association of the sperm with the epithelium of this organ, where sperm heads are inserted into epithelial infoldings and even secretory ductules. The sperm seem to exhibit an affinity for the secretions in these ducts, likely through chemotaxis or similar mechanisms [2,22]. Also, in the lumen of the spermatheca, sperm are frequently associated with secretions [26]. Hence, when attracted to these pockets formed by cuticular infoldings, spermatozoa could secure their attachment to the organ and prevent their elimination during the passage of the oocytes. This could also serve to retain sperm with better fitness while the female reproductive system degrades and absorbs the less fit cells unable to attach themselves to the epithelium. Further works may provide a precise mechanism for this sperm selection.

**Figure 9.**
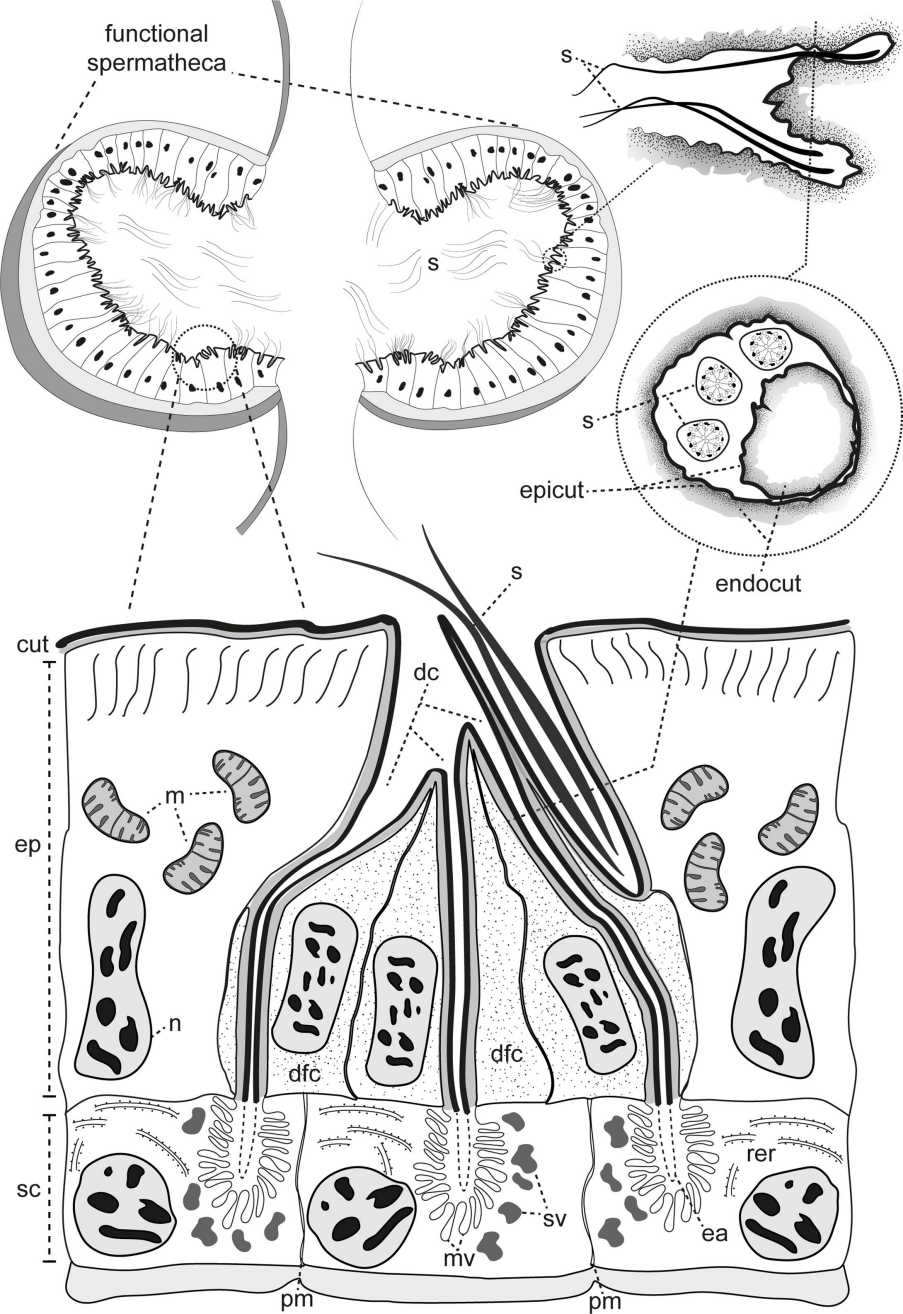
Schematic drawing of the functional spermatheca in Epilachninae. Not to scale. cut: cuticle; dc: ducts; dfc: duct-forming cell; ea: end apparatus; endocut: endocuticle; epicut: epicuticle; ep: epithelial cells; m: mitochondria; n: nucleus; pm: plasma membrane; s: spermatozoa; sc: secretory cells; sv: secretory vesicles; rer: rough endoplasmic reticulum.

An additional and intriguing observation concerns the secretory ducts, which appear to fold inward into their own structure (figure 9). This peculiarity is evidenced by an organization resembling a duct within a duct, where the inner duct displays an inverted cuticle arrangement, with epicuticle and endocuticle orientations opposite to those of the main duct. This morphology has not been observed before for type 3 glandular units [3,4,6]. We assume the arrangement found here is a variation of these class 3 glands, and it could stop sperm from going all the way up the secretory ducts, which could clog them. In *Anastrepha* fruit flies, sperm heads were also observed entering the ductules of the spermathecal glands [15]; however, in this case, without any peculiar modified morphology as reported here.

The other structures in the female reproductive apparatus of *E. clandestina* retain a commonly observed organization. The gland accompanying the vestigial spermatheca shows a typical appearance. It possesses many class 3 secretory cells [2,6], in which rough endoplasmic reticulum and secretory vesicles are observed; however, it did not show a significant secretion amount in its lumen. The epithelium of the vestigial spermatheca did not show any sign of secretory activity, just as no pores were observed in the thick cuticle that covers it, through which secretions could be released, as occurs when this structure is functional [2,6].

In conclusion, the reported secondary spermatheca in *E. clandestina* provides significant insights into the evolutionary adaptations of insect reproductive structures. This differentiated region of the oviduct not only serves to store sperm, optimizing the reproductive capacity of this species but also demonstrates a unique morphology and secretory functionality that challenge traditional classifications of spermathecae. The close association between sperm and the epithelial structures of this spermatheca suggests advanced mechanisms for sperm retention and support, potentially enhancing reproductive success. Furthermore, the findings raise questions regarding the evolutionary trajectory of reproductive adaptations in Epilachninae and other taxa, emphasizing the necessity for a refined understanding of spermathecae across diverse organisms. Future research should focus on the implications of these structures on the evolutionary biology and reproductive physiology of this important insect group.

## Data Availability

This article has no additional data.
